# The oxidative stress and metabolic response of *Acinetobacter baumannii* for aPDT multiple photosensitization

**DOI:** 10.1038/s41598-022-05650-9

**Published:** 2022-02-03

**Authors:** Ewelina Wanarska, Karolina Anna Mielko, Irena Maliszewska, Piotr Młynarz

**Affiliations:** 1 Department of Organic and Medicinal Chemistry, Faculty of Chemistry, Wrocław University of Science and Technology, Wrocław, Poland; 2 Department of Biochemistry, Molecular Biology and Biotechnology, Faculty of Chemistry, Wrocław University of Science and Technology, Wrocław, Poland

**Keywords:** Biotechnology, Computational biology and bioinformatics, Microbiology, Environmental sciences

## Abstract

The use of antimicrobial photodynamic inactivation as a non-antibiotic alternative method to inactivate *Acinetobacter baumannii* was described in response to the ever-growing problem of antibiotic resistance. It was found that irradiation of the bacterial suspension for 10 min reduced the number of viable cells by approximately 99% and this energy fluence was considered to be sub-lethal phototherapy. The lethal dose of laser light (cell mortality about 99.9%) was 9.54 J cm^−2^, which corresponds to 30 min of irradiation. After a 15-fold phototherapy cycle, the tolerance to aPDT decreased, resulting in a decrease in the number of viable cells by 2.15 and 3.23 log_10_ CFU/ml units with the use of sub-lethal and lethal light doses, respectively. Multiple photosensitizations decreased the biofilm formation efficiency by 25 ± 1% and 35 ± 1%, respectively. No changes in antibiotic resistance were observed, whereas the cells were more sensitive to hydrogen peroxide. Metabolomic changes after multiple photosensitization were studied and ^1^H NMR measurements were used in statistical and multivariate data analysis. Many significant changes in the levels of the metabolites were detected demonstrating the response of *A. baumannii* to oxidative stress.

## Introduction

According to data shown by the World Health Organization (WHO), human infections caused by antibiotic-resistant pathogens affect more than 2 million people resulting in over 20,000 deaths annually in the United States and the European Union^1^. Bacterial resistance to anti-microbial agents is known to be growing rapidly, but the rate of development of new antibiotics has decreased significantly over the past three decades^1,2^. WHO has warned that the "post-antibiotic" era in which widespread infections could be fatal to humans is an increasingly real threat^3^.

*Acinetobacter baumannii* is a saprophytic bacterium, widely distributed in the natural environment, including water, soil, sewage and skin of animals and humans^4^. In the past, it was considered a low-category pathogen but today this coccobacillus is one of the major pathogens responsible for causing nosocomial infections, especially in intensive care units (ICUs) worldwide. The organism's ability to contaminate hospital surfaces is associated with hospital outbreaks in which not only hospitalized patients are infected but also the general population. Infections with these bacteria in hospital settings result in a mortality of 26%, which increases to as much as 43% in intensive care units^5^. It has been confirmed that *A. baumannii* is resistant to many classes of antibiotics and disinfectants due to chromosome-mediated genetic elements and may persist for a long time under harsh conditions^6,7^.

*Acinetobacter baumannii* can be easily transmitted through the vicinity of affected patients or colonizers such as curtains, linens fomites, bed rails, tables, sinks, doors, feeding tubes, and even medical equipment. Contamination of respiratory support equipment, suction devices, and devices used for intravascular access is the key source of infection^8^.

One of the methods of effective pathogen inactivation that has gained great interest in many medical communities is antimicrobial photodynamic therapy (aPDT). APDT is not a new technique as it has been used for over 1000 years in Egypt, India, and China^9^, and today it has found application particularly in periodontal infection, endodontic disinfection, and the treatment of localized infections from the dermatological origin^10,11^. This treatment has a significant advantage over existing antimicrobial therapies as it is faster compared to other antimicrobials, equally effective in killing both multi-drug resistant and non-resistant pathogens, and eliminates the secreted virulence factors^12,13^. One of the important advantages of this antimicrobial technique is that it can be applied topically to avoid systemic side effects.

The mechanism of aPDT is based on the absorption by cells of a non-toxic dye called photosensitizer (PS) (the dye can also act on the cell surface), then its activation by visible light of a specific wavelength in the presence of oxygen, causing severe damage to biomolecules and metabolic pathways in the membrane, plasma and genetic material of microbial cells^14^. There are two mechanisms by which, in the presence of oxygen, activation of the photosensitizer to a triple-state can enter into chemical reactions with biomolecules. Type I reactions lead to the generation of free radicals by hydrogen or electron transfer. These reactive species by interacting with oxygen can produce highly reactive oxygen species (ROS) such as superoxide or superoxide anions, which are effective in destroying cells^15^. In a mechanism called Type II, an excited and highly reactive state of oxygen (singlet oxygen) is produced, which is believed to be the major route of killing pathogenic cells^16^. In many cases, it is difficult to distinguish between these two reaction mechanisms and it has already been shown that the mechanism of cell damage depends on the oxygen tension and concentration of photosensitizer^16^.

It should also be emphasized that the non-specific nature of ROS-induced cell death and the short exposure time to PS hinder the expression of protective factors, and bacterial resistance to aPDT is highly unlikely^14^. However, a few years ago, it was demonstrated that aPDT tolerance can develop in Gram-positive bacteria (*S. aureus* MRSA and MSSA) and other cocci, such as *Enterococcus faecium* and *Streptococcus agalactiae*^17,18^. The authors observed an increased frequency of mutations in *S. aureus* after sub-lethal phototherapies, indicating that increased aPDT tolerance may be due to accumulated mutations. Moreover, it was found that aPDT tolerant *S. aureus* was characterized by a higher sensitivity to gentamicin and doxycycline, which supports the hypothesis of genetic alterations induced by sub-lethal phototherapy^17^. QRT-PCR analysis revealed that 10 sub-lethal exposures to aPDT led to increased expression of all major elements of the oxidative stress response (sodA, ahpC, npx, cylE, tpx, and recA)^18^.

Oxidative stress is defined as the disturbance of the balance between the formation of free radicals and antioxidants in favor of oxidants^19^. This imbalance leads to cell damage at the molecular level and it is well known that complex biochemical mechanisms are needed to regulate the entire process^20^. Microorganisms can use a variety of strategies, including increased production of enzymes such as catalases, superoxide dismutases, and the formation of oxidant scavengers (glutathione, pigments, etc.) to avoid cell damage caused by ROS^21,22^. In addition, bacteria exposed to ROS are thought to reorganize important metabolic pathways. Modulation of cellular metabolism under oxidative conditions can be observed by metabolomics. This powerful tool has been used to study the response to oxidative stress in microorganisms and plants^23^. For example, Wirgot et al.^24^ observed that H_2_O_2_ modulated the metabolic functioning of microorganisms in cloud water, as evidenced by strong correlations between H_2_O_2_ and ATP concentrations in cells. In another study, it was demonstrated that *Pseudomonas graminis* after exposition to H_2_O_2_ and radicals had a distinct metabolome as compared to unexposed cells and modulations of certain metabolic pathways in response to oxidative stress were observed^25^. The obtained data indicated that these regulations mainly concerned carbohydrate, glutathione, energy, lipid, peptides, and amino-acids metabolisms^25^. Fountain et al.^26^ reported that *Aspergillus flavus* grown in a medium supplemented with H_2_O_2_ showed extensive stimulation of antioxidant mechanisms and pathways, including polyamine metabolism, glutathione metabolism, TCA cycle, and lipid metabolism.

This study was aimed at assessing whether the application of multiple sub-lethal and lethal phototherapies against *Acinetobacter baumannii* could result in increased tolerance of this coccobacillus to photosensitization. The metabolomics approach was applied to study the pathogen's response to multiple exposures to aPDT. The NMR analyzes identified metabolites that accumulated at different times to elucidate the metabolic pathways influenced by oxidative stress induced by aPDT.

## Results

Our studies were preceded by the determination of the dark antimicrobial activity of Methylene Blue (MB) on planktonic cells of *A. baumannii* and representative results obtained by BacTiter-Glo™ Microbial Cell Viability Assay were collected in Fig. S1 (*Supplementary Information*). As shown in Fig. S1, the bactericidal activity of MB depended on the concentration of this dye, and the values of cell viability reduction were 15.0 ± 0.5, 39.8 ± 0.5, 82 ± 0.5 and 94.0 ± 0.6% for the MB concentration of 12.5, 25, 50, 100 mgL^−1^, respectively (*p* < 0.05). From these results, it was concluded that MB up to a concentration of 12.5 mgL^−1^ caused an insignificant reduction in the number of cells and this dye concentration was used in all subsequent experiments.

To determine the presence of endogenous laser light-activated photosensitizers, the effect of laser light alone on the viability of *A. baumannii* was examined. Bacterial cells were irradiated with laser light for 10 min (energy fluence 3.18 J cm^−2^), and 30 min (9.54 J cm^−2^), and no changes in the number of viable bacteria were observed (data not shown). The results presented in Fig. S2 (*Supplementary Information*) showed that exposure of the tested bacteria with MB as a photosensitizer to laser light for 10, 20, and 30 min resulted in a reduction of *A. baumannii* viability by 1.91 log_10_, 2.86 log_10_ and 3.12 log_10_ units, respectively (*p* < 0.05). Based on these results it was established that irradiation of the bacterial suspension for 10 min reduced the number of viable cells by approximately 99% (~ 2 log_10_ unit reduction) and this energy fluence was considered to be sub-lethal phototherapy. The lethal dose of laser light (cell mortality about 99.9%; 3 log_10_ unit reduction) was 9.54 J cm^−2^, which corresponds to 30 min of irradiation.

### The effects of multiple photosensitization of *A. baumannii*

The suspension culture of *A. baumannii* was exposed to laser light at sub-lethal and lethal doses at daily intervals, and the sensitivity of this microorganism to aPDT with MB as a photosensitizer was assessed after 1, 5, 10, and 15 treatments (Table 1). This multiple photosensitization effect was expressed as a log reduction in the number of bacterial cells.

**Table 1:** The effect of multiple photosensitization on the reduction in the number of *A. baumannii* cells.

Number of exposures to aPDT				
Log reduction R = log_10_(N_0_) − log_10_(N_x_)				
Dose of laser light	1	5	10	15
Sub-lethal	1.91	2.09	1.98	2.15
Lethal	3.12	3.07	3.12	3.23

N_0_ is the number of viable cells before treatment; N_x_ is the number of viable cells after treatment.

As shown in Table 1, the exposure of *A. baumannii* to the sub-lethal dose of laser light showed a reduction in the number of bacteria by 1.91 log_10_, 2.09 log_10_, 1.98 log_10_ and 2.15 log_10_ CFU/ml units after 1, 5, 10, and 15 treatments (*p* < 0.05). In the case of the lethal dose, it was observed that after 1, 5, 10, and 15 irradiations with laser light, the number of cells decreased by 3.12 log_10_, 3.07 log_10_, 3.12 log_10_ and 3.23 log_10_ CFU/ml units (*p* < 0.05). It seems that multiple photosensitization of these bacteria slightly increased the sensitivity of this microorganism to the phototoxic effect of MB.

The next set of our experiments was the study on the effect of multiple photoinactivation of *A. baumannii* on the sensitivity of these bacteria to antibacterial agents. This effect was expressed as MIC values of gentamicin, streptomycin, nalidixic acid, chloramphenicol and hydrogen peroxide. As can be seen in Table S1 (*Supplementary Information*), the MICs of gentamicin and streptomycin against *A. baumannii* were recorded at 6.25 and 12.5 mgL^−1^, respectively. The MIC values of nalidixic acid and chloramphenicol were found at 50 mgL^−1^. These MIC values of antibiotics did not change after the first, fifth, tenth, or even fifteenth of sub-lethal and lethal phototherapies. In the case of hydrogen peroxide, it was found that after fifteen irradiations, the bacterial cells became more sensitive to this agent, and the MIC value decreased from 5.0 gL^−1^ to 1.25 and 0.625 gL^−1^ after exposure to sublethal and lethal doses of light, respectively.

Then, the effect of multiple photosensitization of *A. baumannii* on the efficiency of biofilm formation was studied using MTT test (Table 2). The decrease in biofilm formation efficiency [%] was estimated by comparing the absorbance of the formazan product (Table 2) with the absorbance of the control (see “Materials and methods”), which was assumed to be 100% biofilm forming capacity.

**Table 2:** The effect of multiple photosensitization on the efficiency of biofilm formation by *A.baumannii.* *Mean value of three replicates.

Number of exposures to aPDT				
Absorbance [A_570_]*				
Dose of laser light	1	5	10	15
Sub-lethal	0.78 ± 0.01	0.78 ± 0.01	0.65 ± 0.01	0.62 ± 0.01
Lethal	0.68 ± 0.01	0.64 ± 0.02	0.56 ± 0.02	0.54 ± 0.02

As can be seen in Table 2, the biofilm formation capacity of *A. baumannii* up to the tenth sub-lethal and lethal light doses decreased by approx. 11 ± 1% and 24 ± 1% compared to the control, respectively (*p* < 0.05). After the fifteenth sub-lethal and lethal light doses, the biofilm formation efficiency significantly decreased by 25 ± 1% and 35 ± 1%, respectively (*p* < 0.05).

### Metabolite analysis

The use of NMR analysis allowed the identification of metabolites that are affected by oxidative stress induced by antimicrobial photodynamic therapy. All assignments were verified using the following databases (KEGG Pathways, PubChem). Information about the chemical shift for each metabolite are available in *Supplementary Information* (Table S2). Tables 3 and 4 summarizes the statistical and multivariate data analysis for 35 identified metabolites. VIP scores were calculated for each compound based on ^1^HNMR signal relative intensities to assess whether the compound was more/less present in the samples after aPDI. VIP scores indicate the relative influence of the corresponding metabolites on the discrimination between the two conditions: (*1)* before and after sub-lethal phototherapies (C10/S10 or C15/S15), (*2*) after ten (S10) and fifteen (S15) sub-lethal aPDTs; (*3*) before and after lethal phototherapies (C10/L10 or C15/L15); (*4*) after ten (L10) and fifteen (L15) lethal aPDTs. Metabolites with a VIP score above 1.00 and statistical significance after *p*-value adjustment (*p *value < 0.05) were analyzed. Most of the identified VIP metabolites were amino acids, their derivatives or precursors. The multivariate data analysis clearly showed the natural grouping of samples. The PCA score plots for each analyzed comparison are available in *Supplementary Information* (models for a sub-lethal dose of light—Fig. S3; models for a lethal dose of light—Fig. S4).

**Table 3:**
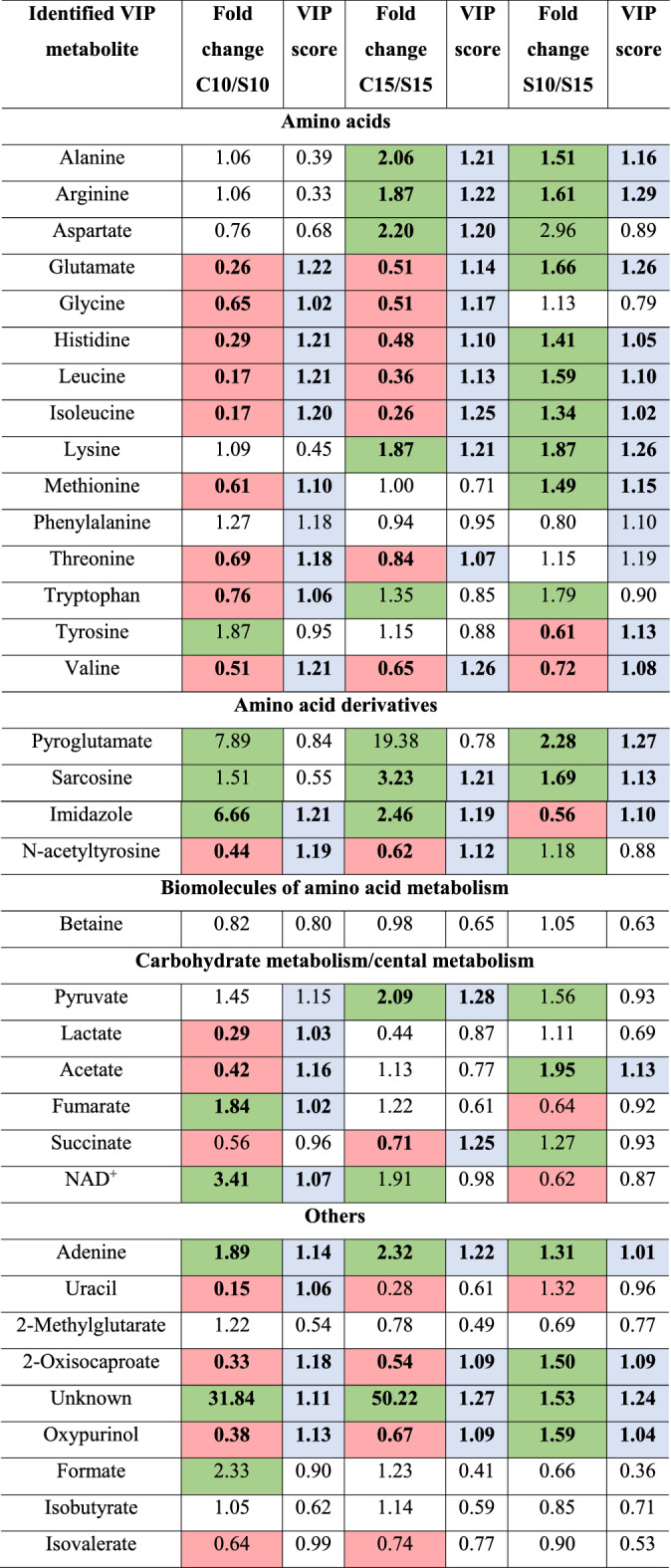
The statistical and multivariate data analysis for identified metabolites before and after multiple sub-lethal exposures of *A. baumannii* to aPDT (increased levels of metabolites with *p* value < 0.05 are marked in red; decreased levels of metabolites with *p* value < 0.05 are marked in green; VIP values > 1.00 are marked in grey; metabolites with *p* value < 0.05 and VIP value > 1.00 are bolded).

C10 or C15- cultures before phototherapies, after ten or fifteen inoculations (controls).

S10 or S15- cultures after ten or fifteen sub-lethal aPDTs.

**Table 4:**
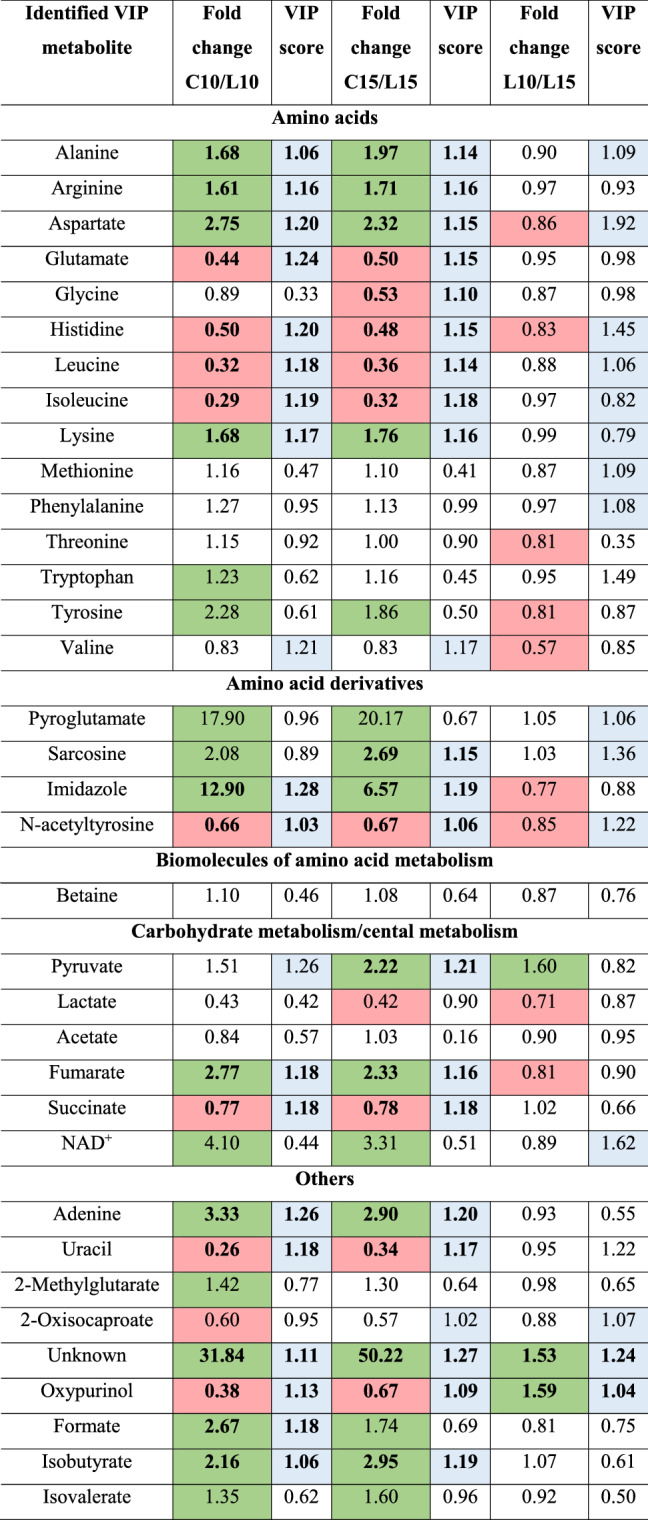
The statistical and multivariate data analysis for identified metabolites before and after multiple lethal exposures of *A. baumannii* to aPDT (increased levels of metabolites with *p* value < 0.05 are marked in red; decreased levels of metabolites metabolites with *p* value < 0.05 are marked in green; VIP values > 1.00 are marked in grey; metabolites with *p* value < 0.05 and VIP value > 1.00 are bolded).

C10 or C15- cultures before phototherapies, after ten or fifteen inoculations.

L10 or L15- cultures after ten or fifteen lethal aPDTs.

The first observed effect of oxidative stress was the changes in the level of amino acids in bacterial cells (Tables 3, 4). As can be seen in Table 3, the levels of glutamate, glycine, histidine, leucine, isoleucine, methionine, threonine, tryptophan, and valine significantly increased after ten sub-lethal exposures of *A. baumannii* to aPDT in the presence of MB as a photosensitizer. Similar results of increased levels of glutamate, glycine, histidine, leucine, and isoleucine were observed after fifteen sub-lethal phototherapies. It should be noted that under the same experimental conditions, the concentrations of alanine, arginine, aspartate, and lysine in the cell significantly decreased. When the studied bacteria were irradiated with a lethal dose of laser light in the presence of MB as a photosensitizer, the concentrations of alanine, arginine, asparagine, and lysine decreased significantly after ten and fifteen sub-lethal exposures of *A. baumannii* to aPDT in the presence of MB as a photosensitizer (Table 4). On the other hand, the concentrations of glutamine, histidine, leucine, and isoleucine in bacterial cells increased after ten and fifteen sub-lethal doses of light in the presence of MB. Interestingly, the level of glycine was also increased but only after fifteen lethal doses of light (Table 4).

Changes in the concentration of some amino acid derivatives in bacterial cells as a result of multiple photosensitization were also found. A reduced level of imidazole was detected in *A. baumannii* cells induced by oxidative stress. When these bacteria were exposed to fifteen sub-lethal and lethal doses of light, the reduction in the concentration of sarcosine was detected. On the other hand, the level of *N*-acetyltyrosine significantly increased after ten and fifteen sub-lethal and lethal phototherapies.

The level of some biomolecules related to the central metabolism was also changed after multiple expositions of *A. baumannii* to aPDT. It was shown that lactate and acetate levels increased after ten sub-lethal doses of light. The increased concentration of succinate was found after fifteen sub-lethal exposures of *A. baumannii* cells to laser light. A similar effect was found after lethal phototherapies. On the contrary, a significant reduction of the fumarate concentration after ten sub-lethal and lethal doses of light was observed.

Pyruvate generated during glycolysis, can be converted to lactate using the enzyme lactate dehydrogenase and the coenzyme NADH in lactate fermentation. As can be seen in Tables 3 and 4, the concentrations of this biomolecule significantly decreased in bacterial cells after multiple laser light-induced photooxidations in the presence of a photosensitizer. A significant reduction in the level of NAD^+^ was observed but only after ten sub-lethal doses of laser light.

Adenine and uracil were also VIP biomolecules identified by NMR. Regardless of the phototoxic light dose, the concentration of uracil was increased, while the level of adenine was decreased. Importantly, irrespective of the light dose, the concentration of oxypurinol increased. The concentrations of formate and isobutyrate decreased but only after ten lethal doses of light.

The relative influence of VIP metabolites on the distinction between the tenth and fifteenth of sub-lethal (S10/S15) and lethal (L10/L15) doses of light was also assessed. As can be seen from Table 3, the concentrations of amino acids (alanine, arginine, glutamine, histidine, leucine, isoleucine, lysine, and methionine) were decreased. The exceptions are tyrosine and valine, as the concentrations of these amino acids increased. The level of amino acid derivatives, such as pyroglutamate and sarcosine, decreased significantly, while an increase in the concentration of imidazole was observed. A reduced level of acetate, adenine, 2-oxicaproate, and oxypurinol was detected in *A. baumannii* cells induced by oxidative stress. In the case of lethal light doses, only aspartate, histidine, and N-acetyltyrosine concentrations increased. On the other hand, the level of oxypurinol was decreased (Table 4).

## Discussion

Taking into account the threat caused by *A. baumannii*, it seems obvious that it is necessary to use non-antibiotic methods to combat these bacteria. It is important to describe not only effective protocols for destroying this pathogen but also to understand the mechanisms of metabolic responses of bacteria that may determine the long-term success of therapy.

In the first part of this work, we demonstrated the effectiveness of antimicrobial photodynamic therapy in the fight against *A. baumannii*. It was found that multiple sub-lethal and lethal doses of light in the presence of MB as a photosensitizer slightly increased the sensitivity of these bacteria to phototherapy. We have shown that after the first sub-lethal dose of light, the number of bacteria was reduced by 98.77%, while after the fifteenth phototherapy the number of viable bacteria was reduced by 99.29%. A similar effect was observed with a lethal dose of light. After the first phototherapy, the number of viable bacteria was reduced by 99.92%, while the fifteenth phototherapy resulted in a reduction of the number of living cells by 99.94%.

Surprisingly, it was revealed that multiple sub-lethal and lethal phototherapies did not affect the sensitivity of this pathogen to classical antibiotics (gentamicin, streptomycin, nalidixic acid, and chloramphenicol). These results are inconsistent with the observations of Rapacka-Zdonczyk et al.^17^. The authors have revealed that *S. aureus* exposed to multiple sub-lethal photosensitizations was characterized by a higher sensitivity to gentamicin and doxycycline. On the other hand, the multiple exposures of *A. baumannii* to sub-lethal and lethal doses of light in the presence of MB increased the sensitivity of these bacteria to H_2_O_2_ by a decrease in the MIC value for this aseptic agent from 5.0 gL^−1^ to 1.25 and 0.625 gL^−1^ (after the fifteen exposures to a sub-lethal and lethal dose of light, respectively). Interesting observations were reported by Green et al.^27^, who studied GABA deficient mutant encoding a GABA transaminase and showed that this mutant was more sensitive to H_2_O_2_. In addition, the aΔpaaJKXYI mutant that lacked part of the phenylacetate degradation pathway was more susceptible to killing by this disinfectant. The authors hypothesized that the MumR-regulated pathways could play the key role in defense against ROS. It was observed that the transcription of a number of catabolic pathways, including phenylacetate and GABA metabolism, was downregulated in the ΔmumR strain growing in a rich medium. Mutants lacking gabT GABA transaminase or paaJKXYI phenylacetate degradation genes showed increased sensitivity to ROS, suggesting that the functions of these pathways may play an important role in the response of *A*. *baumannii* to oxidative stress. The paaJKXYI gene cluster encodes part of the operon that converts the aromatic compound phenylacetate, a breakdown product of phenylalanine, into succinyl coenzyme A (succinyl-CoA) and acetyl-CoA and it is possible that the products of GABA and phenylacetate catabolism may be required to repair or regenerate from oxidative damage. It was speculated that the use of these pathways allow *A. baumannii* to bypass other metabolic pathways impaired by oxidative stress, such as those that require enzymes containing iron-sulfur clusters that are critical for many metabolic functions.

There was also found a slight reduction in the biofilm formation capacity of the studied pathogen. The similar results were obtained by Carmello et al.^28^. These authors observed that photodynamic therapy reduced adhesion capacity and biofilm formation of *Candida albicans* from induced oral candidiasis in mice. It is worth noting that although *A. baumannii* infections have attracted the attention of researchers for several years, the virulence factors of this pathogen, including biofilm formation, are not well understood^29^. It was proved that biofilm-forming strains are known to have a modified phenotype compared to the corresponding planktonic bacteria and show differences in gene transcription^30^. The ability of *A. baumannii* to form biofilm is considered as an effective strategy for increasing the survival of bacteria and staying in a hospital environment under stressful conditions. The following molecules have been identified as factors involved in the formation of biofilm by *A. baumannii*: protein (Bap) encoded by the bap gene, outer membrane protein A (OmpA) encoded by the ompA gene, chaperone pilus (Csu), extracellular exopolysaccharide (EPS), two-component system (BfmS/BfmR), poly-β-(1,6)-*N*-acetylglucosamine (PNAG) and the quorum sensing system. At this stage of the research, we are not able to identify the metabolic changes of *A. baumanni*, which are responsible for the reduced ability of these bacteria to form a biofilm. This phenomenon requires further studies to be carried out in our laboratory.

**Figure 1:**
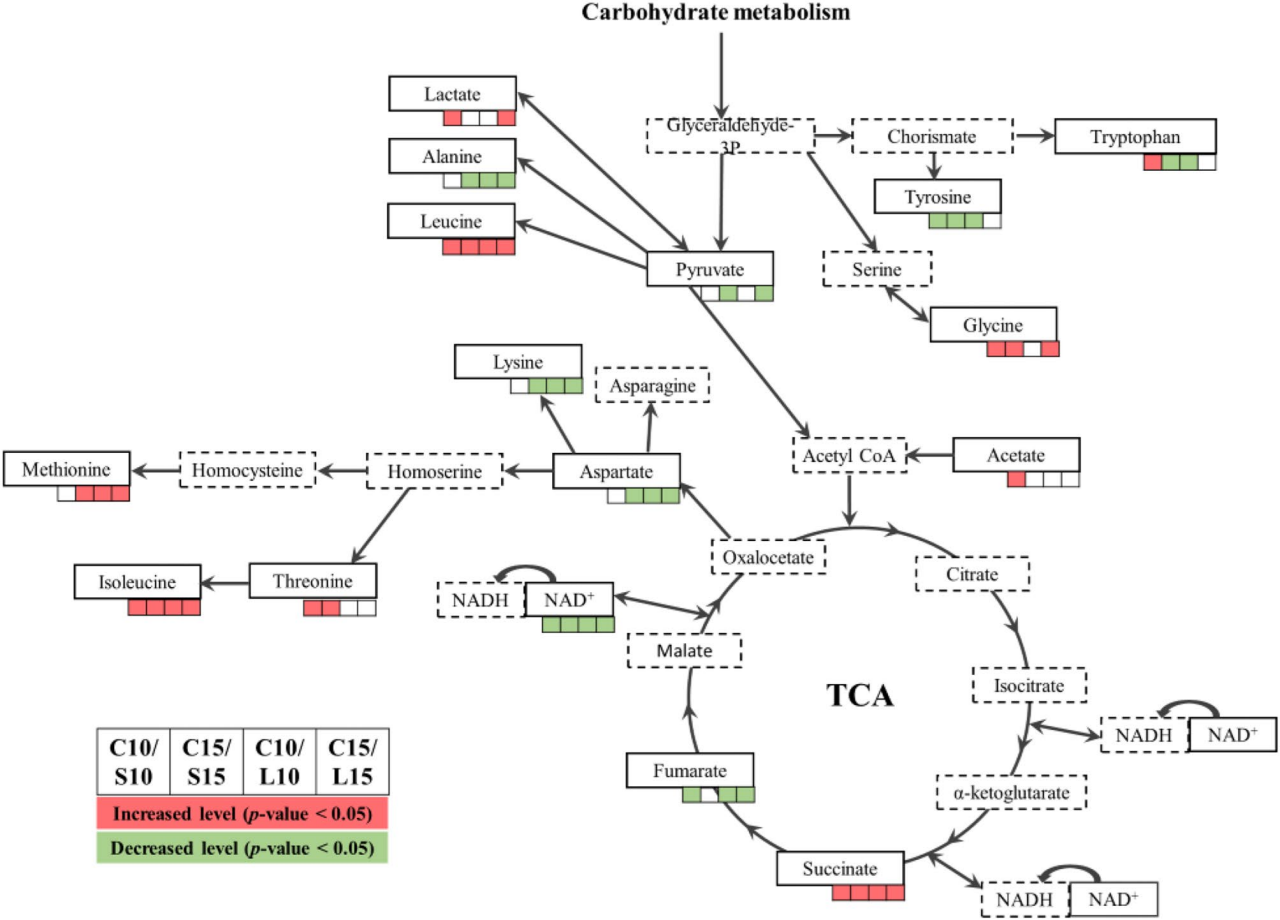
Central metabolism. Green and red indicate significant decreases and increases in metabolite levels, respectively (*p *value < 0.05). The metabolites indicated in the dashed boxes are putative.

In the second part of this work, the metabolomic approach was used to investigate the response of *A. baumannii* to multiple exposures to sub-lethal and lethal doses of laser light in the presence of MB as a photosensitizer. VIP metabolites indicated differences between control samples (designed C10 and C15) and samples after sub-lethal light doses (designed S10 and S15), and after lethal light doses (designed L10 and L15). These metabolites have been grouped into several super pathways, with the majority of the metabolites classified as metabolic reactions of amino acids to oxidative stress. These observations are generally consistent with the findings of other authors^25,26^, however, in our studies we have not confirmed the significant role of fatty acids in the cell's response to oxidative stress. Figure 1 presents changes in the accumulation of biomolecules involved in the central metabolism after multiple photosensitization of *A. baumannii*.

Changes in the concentration of VIP metabolites (Tables 3, 4, Fig. 1) are the result of complex reactions that have occured in the cell due to oxidative destruction of molecules, as well as the regulation of cellular metabolism related to the antioxidant defense. The most significant perturbations after multiple exposures of *A. baumannii* to aPDT concerned the level of amino acids in bacterial cells. The careful inspection of Tables 3 and 4 revealed two trends in amino acid concentration, depending on the irradiation dose and their multiplication. The first comparison for the sublethal dose shows the difference between the tenth and fifteenth multiplication, where a higher level of major amino acids was observed after the S10 irradiation (Table 3). The exceptions are tyrosine and valine, the levels of which were increased with the S15. The second trend is characteristic for the lethal dose, in which higher repetition of irradiations tended to increase the amino acid concentration (however mostly insignificant) (Table 4).

The increase in amino acid concentration may in part be due to increased protein turnover. One of the reasons may be protein degradation as the result of the elimination of abnormal proteins resulting from oxidative stress or may be interpreted as a way to increase the availability of amino acids needed for the synthesis of proteins important for survival under stress conditions^31^.

It is known that sulfur-containing amino acids (cysteine and methionine) are particularly susceptible to ROS reactivity. Other amino acids such as arginine, lysine, proline, and threonine can be carbonylated and histidine can be modified to oxo-histidine^32^. The observed decrease in the concentration of arginine and lysine (Tables 3, 4), regardless of the irradiation dose, may be the result of carbonylation of these amino acids. On the other hand, this change in the concentration of arginine may be related to the easy conversion to ornithine, and then to putrescine. N-acetylputrescine is a part of butanoate metabolism and is used for the biosynthesis of gamma-aminobutanoate (GABA) (https://www.genome.jp/kegg-bin/show_pathway?acb00220). While most of the research on the metabolic role of GABA has focused on its role in carbon and nitrogen metabolism, there is evidence to suggest that it plays a much broader role in bacteria and has a significant impact on survival under environmental stress^33^. The arginine metabolism is also coupled with glutamate, which in contrary to the arginine level is overproduced (https://www.genome.jp/kegg-bin/show_pathway?acb00220).

It is worth to mentioning that the increased concentration of some amino acids and their derivatives may fulfill the antioxidant functions inside the cells. The levels of tryptophan, tyrosine and long-chain acylated tyrosine (Table 3) increased and it cannot be excluded that this phenomenon was related to the inhibition of lipid peroxidation by these amino acids^34,35^. Sulfur-containing amino acid—methionine may also be accumulated in response to oxidative stress after ten expositions of *A.baumannii* cells to the sub-lethal doses of laser light (Table 3). Methionine has antioxidant properties and also acts in important signaling systems^36^. This amino acid contributes to maintain the redox status in cells by supplying the junction metabolite homocysteine as a substrate to the transsulfuration pathway^36^. Next homocysteine can be converted to cysteine, which is incorporated into the antioxidant (glutathione). Interestingly, the decreased level of this amino acid indicated the distinction between the two states S10 and S15 and could be the result of the oxidizing properties of ROS produced during light irradiation of bacteria, or decreased cell metabolism which is weakens after light action. The same role is assigned to cysteine, but this amino acid was not detected as a VIP metabolite in our experiments.

The increased level of glutamate was found in *A. baumannii* cells induced by multiple oxidative stress (Tables 3, 4). It was previously suggested that the activity of the glutamate catabolic pathway (GAD system and GABA shunt) promotes bacterial resistance to numerous stresses^37^. This system facilitates intracellular pH homeostasis by consuming protons in the decarboxylation reaction that produces γ-aminobutyrate (GABA) from glutamate. It seems that role of the GAD system in oxidative stress is related to its influence on intracellular pH. It is well known that the oxidation reactions are influenced by pH, and therefore the increase in pH achieved by the GAD system can change the reaction path and the oxidation processes^38^. It has also been suggested that the NADH or NADPH produced by succinate during GABA catabolism affects redox changes in the cell^38^.

It is worth emphasizing that the increased concentration of glycine and glutamate may result from the fact that these amino acids (along with cysteine) are components of the tripeptide-glutathione that has a radical scavenging function (https://www.genome.jp/dbget-bin/www_bget?pathway:acb00480).

The increased concentration of leucine in the cells of the studied coccobacillus after multiple sub-lethal and lethal photooxidations was also found. It is known that leucine can be used as an alternative source of carbon and nitrogen^38^. L-leucine can be assimilated by bacteria when the sugar metabolism pathways may be "inconvenient" for maintaining cell homeostasis. The main product of these subsequent reactions is 3-methylcrotonyl-CoA metabolite, which undergoes successive enzymatic steps towards the production of acetoacetate and acetyl-CoA^38^. A decreased level of this amino acid indicated a distinction between the two states S10 and S15.

It is assumed that reduced concentration of imidazole after multiple photosensitization of cells may be related to its conversion to glutamate, which plays an important role in protecting the cell against the harmful effects of oxidative stress^37^. An important antioxidant defense mechanism is the production of various bacterial sensory proteins the recognize the cellular redox state and convert chemical information into structural signals to regulate downstream signaling pathways. For example, redox sensors recognize the redox state using amino acids such as cysteine, methionine, and histidine or related cofactors ([Fe-S], heme, flavin, and pyridine nucleotides of compounds involved in the redox process). It can therefore be assumed that high levels of methionine, histidine, and uracil may be associated with these metabolic functions^39^.

The results presented above confirm that changes in concentrations of amino acids are closely involved in antioxidant defense, but the key markers characteristic of *A. baumannii* reaction to multiple photosensitization have not been found. Wirgot et al.^24^ showed that the S/R ratios of valine and tryptophan were particularly high (4.5 and 6.98, respectively) compared to other amino acids with a VIP score (around 2.0) and it was considered to be key markers of *Pseudomonas graminis* response to H_2_O_2_ stress. It should be emphasized that particularly high amino acid ratios in *A. baumannii* cells under no circumstances were observed.

A significant decrease in glycolytic compound pyruvate after multiple photooxidation by sub-lethal and lethal doses of laser light was found. Pyruvate is considered a key intermediate in the oxidative or anaerobic glucose metabolism, but it is also a powerful and effective ROS scavenger (https://www.genome.jp/dbget-bin/www_bget?pathway:acb00620)^40^.

Previously it was revealed that the tricarboxylic acid cycle may be tweaked to limit the formation of NADH. This metabolic redox-balancing act appears to afford a potent tool against oxidative challenge and may be a more widespread ROS-combating tactic than hitherto recognized^25^. The obtained results demonstrated the significant reduction of fumarate in *A. baumannii* cells in response to oxidative stress. This metabolic act of redox balancing appears to be a powerful tool against oxidative stress^41^.

Succinate is another important intermediate product of the tricarboxylic acid cycle under normoxic conditions and glutamine-dependent anaplerosis or γ-aminobutyric acid leakage under anaerobic conditions. The increase in succinate concentration after multiple expositions of bacterial cells to sub-lethal and lethal doses of laser light in the presence of MB was observed. This compound is usually considered an intermediate product but its accumulation was also observed during metabolic stress^42^.

Changes in concentrations of two important metabolites-lactate and acetate were also observed. Lactate can be used by cells as an oxidizing substrate, or it can be oxidized to produce ATP. The increase in lactate concentration in *A. baumannii* cells may be surprising as it has recently been shown to induce ROS^43^. On the other hand, Tauffenberge et al.^44^ reported that the increase in lactate concentration promotes stress resistance by activation of pro-oxidative mechanisms. According to the authors, this finding is consistent with the concept of mitohormesis described in many organisms, in which a moderate increase in ROS production promotes stress resistance by the activation of unfolded protein responses and detoxification mechanisms. It should be noted that lactate promotes resistance to oxidative stress and pyruvate restores the lactate-mediated protective effect. An increased level of acetate indicated a distinction between the two states C10 and S10. Acetate is ubiquitous in the environment and is one of the major short-chain fatty acids excreted by cells in a process called acetate overflow^45^. Acetate can be produced directly from pyruvate by enzymatic decarboxylation or from acetyl-CoA via an intermediate acetylphosphate (reactions catalyzed by the enzymes phosphotransacetylase and acetate kinase). A high concentration of intracellular acetate can reduce the level of the major intracellular anion, i.e. glutamate. It has been noticed that the presence of acetate modulates the expression of genes involved in various biological functions: mobility, biofilm formation, translation, stress response, metabolism, carbohydrate transport, amino acid ions.

The concentration of NAD significantly decreased after ten exposures of *A. baumannii* to sub-lethal photodynamic therapy, which may have been due to fine-tuning the redox potential of cells (NAD^+^/NADH ratios). As multiple photosensitization of cells affects glutathione metabolism, glycolysis, the TCA cycle, and the DNA repair system, it seems understandable that it may also indirectly affect the NAD^+^/NADH coenzymes. This phenomenon may be related to metabolic modulations favoring the production of NADPH, which is involved in many anti-oxidant enzymatic reactions^24^ and it is worth noting that it did not occur after fifteen sub-lethal light exposures or after exposure to lethal phototherapy.

As can be seen in Tables 3 and 4, the damage caused by ROS may also increase uracil concentration. Liu et al.^46^ found that oxidative stress had a stronger effect on RNA. Additionally, they showed that highly structured RNAs like tRNA and rRNA are not protected against oxidative damage. This observation is consistent with the findings of other groups that oxidative stress generally inhibits the translation process^46^.

In summary**,** we have demonstrated that antimicrobial photodynamic therapy can lead to the effective killing of *A. baumannii* planktonic culture. It was confirmed that multiple sub-lethal and lethal doses of light in the presence of MB as a photosensitizer slightly increased the sensitivity of these bacteria to phototherapy and decreased their biofilm formation capacity. Moreover, it was revealed that multiple sub-lethal and lethal phototherapies did not affect the sensitivity of this pathogen to classical antibiotics but increased its sensitivity to H_2_O_2_. It was clearly shown that multiple photooxidations directly impact the metabolome of *A. baumannii* and many metabolic pathways are modified. It needs to be highlighted that changes in the level of some metabolites suggest that prolonged exposures of *A. baumannii* to sub-lethal and lethal doses of light may result in the breakdown of certain defense systems. For example, the concentration of sarcosine (N-methylglycine) significantly decreased in the bacterial cells and it was supposed that a high level of this metabolite was connected with balancing the redox reactions, thus protecting against oxidative damage in cells^47^.

Understanding the metabolic changes induced by multiple photosensitization of *A. baumannii* and the cell defense systems remains an open question, requiring further analysis of complex metabolic pathways, also at the level of gene expression.

## Materials and methods

### Reagents

Chemicals used in this research were obtained from POCH (Poland), Sigma Aldrich (Poland), and Promega (Promega GmbH, Germany). Gentamycin sulfate salt, streptomycin, nalidixic acid, and chloramphenicol were obtained from PPH Galfarm (Poland). Methylene blue (MB) was dissolved in deionized water to a final concentration of 1 gL^−1^ and sterilized by filtration through 0.22-µm pore diameter membranes (Millex^®^-HP syringe-driven filter unit, Millipore). The solution was stored in dark at room temperature. BacTiter-Glo™ Reagent was prepared by mixing BacTiter-Glo™ Buffer and Substrate. The solution was stored in dark at − 78 °C.

MTT reagent was prepared by dissolving 3-(4,5-dimethylthiazol-2-yl)-2,5-diphenyltetrazolium bromide in PBS (pH = 7.2) to a final concentration of 5 gL^−1^. Acidic isopropanol was prepared using 50 ml of isopropanol and 0.75 ml of HCl.

### Light source

Diode laser with the peak-power wavelength λ = 635 nm (output power of 40 mW; the light intensity of 105 mW cm^2^) was used in this study.

### Culture conditions

The one colony of bacterium *Acinetobacter baumannii* (PCM 8740) was inoculated in 5 ml of Mueller–Hinton Broth. The suspension was incubated for 24 h in dark at 37 ºC. This culture of *A. baumannii* was centrifuged (5 min/6000 rpm) and the obtained pellet was resuspended in sterile deionized water. OD_550nm_ was measured and adjusted to 0.09. Then, serial dilutions were prepared and 100 µl aliquots of each dilution were seeded in duplicate onto Mueller–Hinton Agar and incubated for 24 h in dark at 37 °C. After incubation, the number of colony-forming unit (CFU/ml) was determined. The study was carried out in duplicate.

### Studies on the effect of multiple photosensitizations of *A. baumannii*

**Figure 2:**
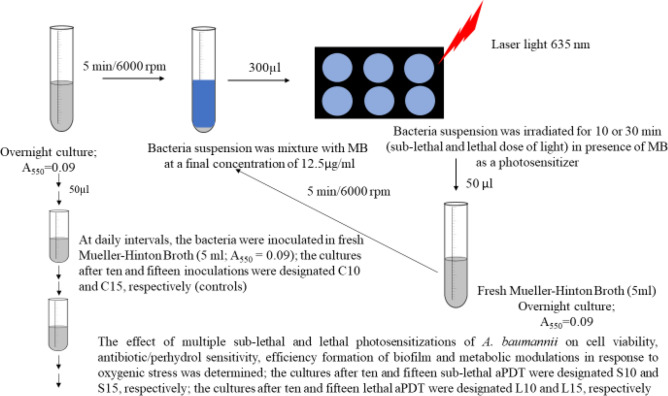
A protocol for conducting multiple sub-lethal and lethal photosensitizations of *A. baumannii.*

The multiple sub-lethal and lethal phototherapy of *A. baumannii* was carried out at 24-h intervals according to the procedure described in Determination of sub-lethal and lethal phototherapy (see *Supplementary Information*) (Fig. 2). The samples were irradiated for 10 or 30 min, which corresponded to the sub-lethal and lethal phototherapy, respectively. Control and irradiated samples in a volume of 0.05 ml were inoculated in 5 ml of fresh Mueller–Hinton Broth, followed by incubation for 24 h in the dark at 37 °C. The overnight cultures were irradiated as initial suspensions for 15 consecutive days under the same conditions. The effect of multiple irradiations of *A. baumannii* with a sub-lethal and lethal dose of light on cell viability, antibiotic/ hydrogen peroxide sensitivity, and efficiency formation of biofilm was examined. The metabolic modulations in response to oxidative stress were determined after 10 and 15 sub-lethal and lethal doses of laser light.

### Determination of susceptibility of *A. baumannii* to antibiotics and hydrogen peroxide

The effect of gentamycin, streptomycin, nalidixic acid, and chloramphenicol at concentrations ranging from 50 to 0.049 mgL^−1^ on the growth of *A. baumannii* was determined by the microdilution method according to the protocol described by Wiegand et al.^48^. In the case of hydrogen peroxide, this study was performed for concentrations ranging from 10 to 0.01 gL^−1^. MIC was defined as the lowest concentration of the antimicrobial agent that prevents visible growth (OD_550_ = 0.0) of *A. baumannii* under defined conditions.

### Effect of sub-lethal and lethal photosensitizations on biofilm formation

Biofilms were formed on 8 mm diameter cellulose nitrate disks. At first, the disks were placed individually in the wells of a microtiter plate and 0.5 ml of a standardized *A. baumannii* suspension (see “Culture conditions”) was transferred into each well and incubated at 37 °C for 4 h to allow for adherence of bacterial cells to the surface of disks. After this time, the supernatant (containing non-adhered cells) was removed and each disc was gently washed using phosphate-buffered saline (PBS). Then, the discs were again placed individually in the sterile wells of a microtiter plate and 0.5 ml of fresh medium was added to each well and the plate was further incubated for 24 h (37 °C). After 24 h biofilm formation, the discs were gently washed with PBS, and the number of viable cells was determined by the MTT test^49^.

The formation of biofilm was measured using the formula:

$$\frac{AT - AB}{{AC - AB}}*100\%$$

where: AC- Absorbance of control sample (no irradiated); formazan absorbance values were 0.87, 0.89, 0.73, and 0.82 after 1, 5, 10, and 15 inoculations of bacteria (see Fig. 2). AB- Absorbance of background sample (no cells, 0.018). AT- Absorbance of the tested sample (irradiated with laser light).

### Extraction and samples preparation for analysis of the bacterial metabolites

The cultures prepared according to the protocol described above (see Fig. 2) were centrifuged (5 min/6000 rpm) and washed in deionized water. All samples were lyophilized and were used for the analysis of the bacterial metabolites. 14.5 mg of lyophilized cells were suspended in 0.7 ml of methanol and samples were disrupted for 5 min in Tissue Lyser (Tissue Lyser II, Qiagen). Then 0.7 ml of water were added to each sample and again vortexed for 10 min. After the disintegration, samples were centrifuged (10 min/12,000 rpm/4 °C) (Micro 220R, Hettich), and 1.1 ml of clarified upper phase was transferred into a new tube. The extracts were evaporated in a vacuum centrifuge (8 h/1500 rpm/40 °C) (WP-03, JW Electronic). In the next step, 0.6 ml of PBS buffer (0.5 M, 10% D_2_O, pH = 7.0, TSP = 0.15 mM) were added to each sample and mixed for 1 min and 0.55 ml were transferred into 5-mm NMR-tubes (5SP, Armar Chemicals) for measurements. Until the measurements ware taken, the samples were stored at 4 °C. The analysis of bacterial metabolites was performed in ten replicates.

### ^1^H NMR spectroscopy analysis of the bacterial metabolites

Standard ^1^H NMR experiments were performed on a Bruker AVANCE II 600.58 MHz spectrometer equipped with a 5 mm TBO probe at 298 K. All one-dimensional ^1^H NMR spectra were carried out using the cpmgpr1d (in Bruker notation) pulse sequence by suppression of water resonance by presaturation. Acquisition parameters were as follows: spectral width, 10 ppm; the number of scans, 128; acquisition time, 2.72 s per scan; relaxation delay, 3.5 s; and time-domain points, 64 K. The spectra were referenced to the TSP resonance at 0.0 ppm and manually corrected for phase and baseline (MestReNova v. 11.0.3).

### Data processing and multivariate statistical data analysis

All spectra were exported to Matlab (Matlab v. 8.3.0.532) for pre-processing. Regions affected by solvent suppression were excluded (4.54–5.12 ppm) and alignment procedures involving the correlation of optimized warping (COW) and interval correlation shifting (icoshift) algorithms were applied^50,51^. The spectra consisted of 8 910 data points and were normalized using the probabilistic quotient method to overcome the issue of dilution^52^. The multivariate and statistical data analysis was performed on a set of the 35 assigned metabolites. The concentration of metabolite measured by NMR was obtained as the sum of the intensities of the no overlapping resonances (or a part of partly overlapping resonances). The input for SIMCA-P software was a transformed data matrix (v 15.02, Umetrics, Umeå, Sweden). For additional analysis, Matlab was used. The data sets were unit variance scaled before the chemometric analysis. For bacteria strains classification, principal component analysis (PCA), and partial least square analysis (OPLS) were carried out. The OPLS-DA model reliability was tested with CV-ANOVA at the level of significance of α < 0.05. Univariate analysis was performed by the use of MATLAB software (v R2019a, Mathworks Inc.) with the use of Student s t-test (equal/unequal variance) for data originated from a normal distribution and using Mann–Whitney–Wilcoxon test for data that does not meet these requirements. Normality of distribution was assessed by the Shapiro–Wilk test. The correction for multiple comparisons was preceded with the Benjamini-Hochberg.

## Supplementary Information

Supplementary Information.
