# Supplementary material for: The oxidative stress and metabolic response of Acinetobacter baumannii for aPDT multiple photosensitization
Source: Sci Rep. 2022 Feb 3;12:1913. doi: 10.1038/s41598-022-05650-9 (PMC8814140; doi:10.1038/s41598-022-05650-9)
Supplement: Supplementary file 1 — Supplementary Information. [file 41598_2022_5650_MOESM1_ESM.docx]

**Supplementary Information**

**The oxidative stress and metabolic response of *Acinetobacter* *baumannii***

***for aPDT* multiple photosensitization**

Ewelina Wanarska^1^, Karolina Anna Mielko^2^, Irena Maliszewska^1,*^, Piotr Młynarz^2^

^1^ Department of Organic and Medicinal Chemistry, Faculty of Chemistry, Wrocław University of Science and

Technology, Poland

^2^ Department of Biochemistry, Molecular Biology and Biotechnology, Faculty of Chemistry, Wrocław University

of Science and Technology, Poland

*Correspondence: irena.helena.maliszewska@pwr.edu.pl

**Determination of the effect of Methylene Blue on *A. baumannii* viability (dark cytotoxicity).** The bacterial suspensions obtained according to the protocol described above were mixed with initial solution of MB to final concentrations of 100, 50, 25, 12.5 mgL^-1^. Then these samples were incubated for 2 h in dark at 37^o^C. After incubation time, the number of viable cells were determined using BacTiter-Glo™ Microbial Cell Viability Assay. The fluorescence intensities were measured by SpectraMax Gemini dual-monochromator. The bacterial suspension without MB was incubated under the same reaction conditions and was used as a control. The study was carried out in duplicate.

**Determination of sub-lethal and lethal phototherapy.** The bacterial suspension obtained according protocol described in *Culture conditions* was mixed with MB to a final concentration of 12.5 mgL^-1^ and 0.3 ml of this mixture was added to well of 96-well flat-bottom microtiter plate. The plate was then shaken for 30 min in an orbital shaker (in dark) at the temperature of 37 ºC. After that the mixture of bacteria suspension and MB was irradiated using laser light (635 nm, light intensity of 105 mW∙cm^-1^) for 10, 20 and 30 minutes (control probe was unirradiated bacterial suspension). The number of viable cells was determined using method of series dilution according to the protocol described above.

To evaluate photo-bactericidal effect of MB, the percentage reduction (%) of *A. baumannii* was calculated using the following formula. The reduction in viability R = (N_0_ ̶ N) $\times$ 100/N_0_, where N_0_ and N are the numbers of CFUs at initial and remaining in suspension after laser light irradiation. The sub-lethal phototherapy was considered to be the dose of light that in the presence of MB at a concentration of 12.5 mgL^-1^ as a photosensitizer reduced the viability of bacteria by 2 log_10_ units (99%). The light dose that, under the experimental conditions described above, reduced the viability of the bacteria by 3 log_10_ units (99.9%) was considered to be a lethal phototherapy.

The study was carried out in triplicate.

Figure S1. The effect of Methylene Blue concentration on the viability of *A. baumannii* (dark cytotoxicity)

Figure S2. The effect of exposure time to laser light on the viability of *A. baumannii* with Methylene Blue as a photosensitizer (the concentration of MB was 12.5 mgL^-1^)

Table S1. Minimal inhibitory concentrations of antibacterial agents

| **Number of exposures to aPDT**  **Minimum Inhibitory Concentration [mgL^-1^]** | | | | | |
| --- | --- | --- | --- | --- | --- |
| Antibiotic | 0 | 1 | 5 | 10 | 15 |
| Gentamicin | 6.25/6.25^*^ | 6.25/6.25 | 6.25/6.25 | 6.25/6.25 | 6.25/6.25 |
| Streptomycin | 12.5/12.5 | 12.5/12.5 | 12.5/12.5 | 12.5/12.5 | 12.5/12.5 |
| Nalidixic acid | 50/50 | 50/50 | 50/50 | 50/50 | 50/50 |
| Chloramphenicol | 50/50 | 50/50 | 50/50 | 50/50 | 50/50 |
| Hydrogen peroxide | 5000/5000 | 5000/5000 | 5000/5000 | 5000/5000 | 1250/625 |

^*^sub-lethal dose of light/lethal dose of light

0-without light treatment (control); 1-after one light treatment; 5- after five light treatments; 10- after ten treatments with light; 15-after fifteen treatments with light

Table S2. ^1^H NMR signal assignments.

| **Metabolite** | **KEGG number** | **Chemical shift [ppm]** |
| --- | --- | --- |
| **2-methylglutarate** | C16390 | 1.1 (d) |
| **2-oxisocaproate** | C00141 | 2.6 (d) |
| **unknown** | C02505 | 2.0 (s) |
| **acetate** | C00033 | 1.9 (s) |
| **adenine** | C00147 | 8.2 (d) |
| **alanine** | C00041 | 1.5 (d) |
| **arginine** | C00062 | 3.2 (t) |
| **aspartate** | C00049 | 2.8 (dd) |
| **betaine** | C00719 | 3.9 (s) |
| **formate** | C00058 | 8.4 (s) |
| **fumarate** | C00122 | 6.5 (s) |
| **glutamate** | C00025 | 2.4 (m) |
| **glycine** | C00037 | 3.6 (s) |
| **histidine** | C00135 | 8.0 (s) |
| **imidazole** | C05568 | 8.3 (s) |
| **isobutyrate** | C20846 | 1.0 (d) |
| **isoleucine** | C00407 | 3.7 (d) |
| **isovalerate** | C20827 | 0.9 (d) |
| **lactate** | C00256 | 1.3 (d) |
| **leucine** | C00123 | 1.0 (dd) |
| **lysine** | C00047 | 3.7 (t) |
| **methanol** | C00132 | 3.3 (s) |
| **methionine** | C00073 | 2.1 (s) |
| **N-acetyltyrosine** | C00082 | 6.8 (d) |
| **oxypurinol** | C07599 | 8.2 (s) |
| **phenylalanine** | C00079 | 7.4 (m) |
| **propane-1,2-diol** | C00583 | 1.1 (d) |
| **pyroglutamate** | C01879 | 4.2 (m) |
| **pyruvate** | C00022 | 2.4 (s) |
| **sarcosine** | C00213 | 2.7 (s) |
| **succinate** | C00042 | 2.4 (s) |
| **threonine** | C00188 | 3.6 (d) |
| **tryptophan** | C00078 | 7.7 (d) |
| **tyrosine** | C00082 | 6.9 (d) |
| **uracil** | C00106 | 5.8 (d) |
| **valine** | C00183 | 1.0 (d) |
| **NAD+** | C0000 | 9.2 (d) |


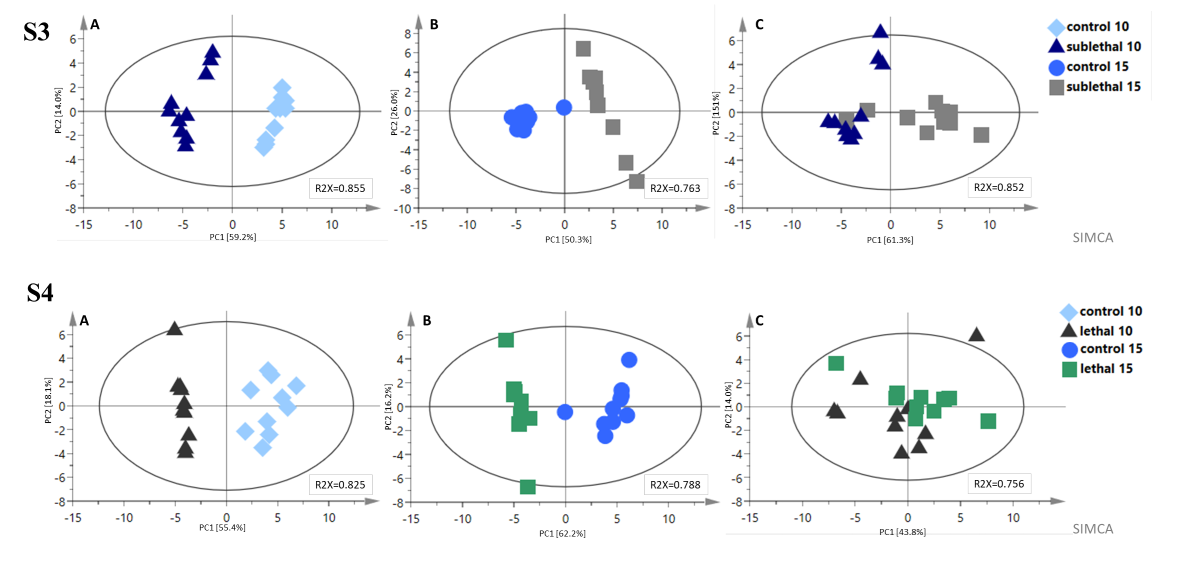


Figure S3. The PCA score plots for the sub-lethal dose of light comparison (A – C10/S10; B – C15/S15; C – S10/S15)

Figure S4. The PCA score plots for the lethal dose of light comparison (A – C10/L10; B – C15/L15; C – L10/L15).
